# Risk of hypertension in women with polycystic ovary syndrome: a systematic review, meta-analysis and meta-regression

**DOI:** 10.1186/s12958-020-00576-1

**Published:** 2020-03-17

**Authors:** Mina Amiri, Fahimeh Ramezani Tehrani, Samira Behboudi-Gandevani, Razieh Bidhendi-Yarandi, Enrico Carmina

**Affiliations:** 1 Reproductive Endocrinology Research Center, Research Institute for Endocrine Sciences, Shahid Beheshti University of Medical Sciences, No 24, Parvane Street, Yaman Street, Velenjak, Tehran, Iran; 2 Faculty of Nursing and Health Sciences, Nord University, Bodø, Norway; 3 School of public health, Department of Epidemiology and biostatistics, Tehran University of Medical Sciences, Tehran, Iran; 4 Endocrinology Unit, Department of Health Sciences and Mother and Child Care, University of Palermo, Palermo, Italy

**Keywords:** Meta-analysis, Hypertension, Polycystic ovary syndrome, Relative risk

## Abstract

**Background:**

A limited number of publications have assessed the prevalence of hypertension (HTN) in polycystic ovary syndrome (PCOS) patients with inconclusive results. Since in general populations the occurrence of hypertension is related to age per se, we investigated the prevalence (P) / relative risk (RR) of HTN in pooled patients with PCOS, vs control population among reproductive age women with PCOS, compared to menopause/aging patients.

**Methods:**

PubMed, Scopus, ScienceDirect, web of science, and Google scholar were systematically searched for retrieving observational studies published from inception to April 2019 investigating the HTN in patients with PCOS. The primary outcome of interest was pooled P and RR of HTN in reproductive and menopausal/aging women with PCOS compared to control population.

**Results:**

The pooled prevalence of HTN in reproductive and menopausal/aging women with PCOS was higher than in the control population [(Pooled P: 0.15, 95% CI: 0.12–0.18 vs. Pooled P: 0.09, 95% CI: 0.08–0.10) and (Pooled P: 0.49, 95% CI: 0.28–0.70 vs. Pooled P: 0.40, 95% CI: 0.22–0.57), respectively]. Compared to the control population, pooled relative risk (RR) of HTN patients was increased only in reproductive age PCOS (1.70-fold, 95% CI: 1.43–2.07) but not in menopausal/aging patients who had PCOS during their reproductive years. The same results were obtained for subgroups of population-based studies. Meta-regression analysis of population-based studies showed that the RR of HTN in reproductive age PCOS patients was 1.76-fold than menopausal/aging PCOS patients (*P* = 0.262).

**Conclusion:**

This meta-analysis confirms a greater risk of HTN in PCOS patients but demonstrates that this risk is increased only in reproductive age women with PCOS, indicating that after menopause, having a history of PCOS may not be as an important predisposing factor for developing HTN.

## Background

Polycystic ovary syndrome (PCOS), a prevalent endocrine and metabolic condition in reproductive age women [1], is a heterogeneous disorder that is associated to increased cardiovascular risk. Previous studies have demonstrated that almost all cardiovascular (CV) risk factors including obesity, insulin resistance, diabetes mellitus, atherogenic dyslipidemia, metabolic syndrome, C-reactive protein are elevated in patients with PCOS [2–6]; these risk factors are present even in young PCOS patients and predispose to development of early atherosclerosis, cardiovascular morbidity and mortality [5, 7, 8].

Androgen excess in patients PCOS is clearly associated with an increased prevalence of cardio-metabolic disturbances [9]. Evidence demonstrated that an increased prevalence of subclinical atherosclerosis, endothelial dysfunction, increased carotid intima media thickness and coronary artery calcification in PCOS patients [6]. Shroff et al. showed a 5-fold higher prevalence of subclinical coronary atherosclerosis in young obese women with PCOS, compared to general population [10]. The prevalence of coronary artery calcification is 4-fold higher than control population (39.0% vs. 9.9%) [11]. It is well documented that PCOS in young women is associated with endothelial dysfunction [4, 12]. Previous studies showed an atherogenic lipid profile and increased plasminogen activator type 1 (PAI-I) production in PCOS patients, which these risk factors are important for developing cardiovascular disease [13, 14].

While hypertension (HTN) represents one of the main cardiovascular risk factors in general populations, only a few studies have investigated HTN in women with PCOS and these have with contrasting results [15–19]. On the other hand, it is not yet known whether increased risk of HTN modifies by aging [19].

To get more information on the prevalence and the evolution of HTN in women with PCOS, we performed a meta-analysis of available data assessing the prevalence and risk of HTN not only in all women with PCOS but also in these patients divided according to their age (reproductive and postmenopausal). In both approaches, population based and non-population-based studies were analyzed separately.

## Materials and methods

This systematic review and meta-analysis was designed according to the guidelines for the Preferred Reporting Items for Systematic Reviews and Meta-Analyses (PRISMA) [20] and the Cochrane Handbook for Systematic Review of Interventions [21] to investigate the pooled prevalence (P) / RR of HTN:

1. In reproductive and menopausal/aging PCOS groups, compared to control population.
2. In reproductive and menopausal/aging PCOS groups compared to control population in population-based studies.
3. Between reproductive age PCOS women compared to menopausal/aging group.
4. Between reproductive age PCOS women compared to those in menopausal/aging group in population-based studies.

### Search strategy

In this meta-analysis, PubMed, Scopus, ScienceDirect, web of science, and Google scholar were searched for retrieving observational studies published from inception to April 2019 investigating the HTN in patients with PCOS.

Before initiation of the study, we conducted the search strategy with the assistance of a professional healthcare librarian. All reviewers performed searches separately. At first, search in the PubMed was performed based on control vocabularies (MESH) using the following formula: (“Polycystic ovary syndrome” OR “PCOS”) AND (“cardiovascular” OR “cardio-metabolic” OR “metabolic” OR “hypertension” OR “hypertensive” OR “blood pressure”).

We also searched PubMed and other databases using free-text terms. Search criteria were humans, and English language. Search strategy was almost similar for all databases. The searches were done based on the ‘all fields’ in the PubMed and ‘titles, abstracts and keywords’ in other databases. A ‘pearl growing’ strategy was employed, whereby, after obtaining the full text articles, the reference lists of all included studies were reviewed for additional publications that could be used in this review.

### Eligibility criteria

Analytic observational studies of all types including cross-sectional, case-control, and cohort designs that assessed hypertension in women with PCOS were eligible to be included in the meta-analysis. In addition, studies needed to report a risk ratio (odds ratio [OR], relative risk [RR], or hazard ratio) or should have provided sufficient information to allow calculation of a relevant effect estimate. We included studies using National Institutes of Health (NIH) [22], Rotterdam [23], Androgen Excess Society (AES) [24], laparoscopic [25] and International Classification of Diseases (ICD) [26] as diagnostic criteria for PCOS.

Exclusion criteria included: (1) studies that did not differentiate between women and men, (2) studies that did not assess any cardiovascular events, (3) studies with unreliable and incomplete results, and (4) studies with unknown or invalid PCOS diagnostic criteria.

### Study selection

We included all relevant studies assessing hypertension in women with PCOS. The results of the searches were screened based on predefined eligibility criteria. All references were entered to EndNote X7 software (Thomson Reuters, New York, NY, USA). Initial selection was performed based on their titles, followed by a second selection performed by one reviewer (M.A), who deleted duplicates and reviewed the abstracts of all remaining records. Any disagreement in the selection of abstracts was resolved by consensus or by two other reviewers (F.R.T and E.C). Full text articles for review and data processing were obtained for all selected abstracts.

### Data extraction

Two reviewers (M.A and S.B.G), in close consultation with another reviewer (F.R.T), extracted data from full text articles; they rechecked all precisely extracted data to minimize errors. For each study, the following information was extracted: Authors, year of publication, title, study design (cross-sectional, case-control, cohort), characteristics of study population, number of events, and the unadjusted or adjusted risk ratios provided (OR, RR, or hazard ratio) by each outcome. To prevent extraction errors, all reviewers performed a quality control check between the final data used in the meta-analysis and the original publications.

### Quality assessment

All studies included for the meta-analysis were appraised for the quality of their methodological and result presentation. Two reviewers (M.A and S.B.G) assessed the quality of the studies separately. They were blinded to study author, institution, and journal name. Disagreement was resolved and adjusted by other reviewers (F.R.T). All observational studies including cross-sectional, case-control, and cohort were appraised according to the Newcastle–Ottawa scale [27]. In this respect, 3 domains were scored for selection and comparability of study cohorts, and determination of the outcome of interest. If a study obtained ≥70% of the highest level of the Newcastle–Ottawa scale, it was considered to be of high quality, 40–70% as moderate, and 20 to 40% as low and < 20% as very low quality (Supplementary File 1).

### Risk of bias assessment

We assessed the risk of bias in each study included, using the Cochrane Collaboration’s tools, which have been designed for various methodological studies including cross-sectional, case-control, and cohorts. Review authors’ judgments were categorized as “low risk,” “high risk,” and “unclear risk” of bias (either low or high risk of bias) [28].

### Outcome measures

The primary outcome of interest was hypertension, which was defined as systolic blood pressure (SBP) ≥ 140 mmHg and diastolic blood pressure (DBP) ≥ 90 mmHg or current use of anti-hypertensive medicine [29].

### Statistical analysis

We performed a meta-analysis to estimate the pooled P / RRs of HTN. Heterogeneity was evaluated using the I-squared (I^2^) statistics; values above 50% were interpreted as heterogeneity. Both random and fixed effect models were used for heterogeneous and non-heterogeneous results, respectively. Publication bias was assessed using the Begg’s test [30]; bias was found to be significant for *P*-values < 0.05. The trim and fill method was not used because of non-significant results [31].

Pooled P and pooled RR (Pooled RR) were used for reporting results of the meta-analysis. The “Meta-prop” method was applied for the pooled estimation of the prevalence of HTN [32]; pooled RR was also estimated by the “Metan” method, using the normal distribution to estimate confidence intervals. Mantel–Haenszel method was used to estimate pooled data [33].

Subgroup analyses were performed to assess the pooled P / RR of HTN, based on age groups (reproductive vs. menopausal/aging) and study design (population- vs. non-population-based studies). Forest plots were drawn for RR of HTN in the mentioned subgroups as well.

Furthermore, the random effect meta-regression model was fitted to estimate the association between age groups and outcome of interest (here RR of HTN) in subgroups of study design. Bubble plots were drawn to illustrate the fitted models for each covariate.

We also adjust BMI and diabetes mellitus as a confounding variable to decrease the source of heterogeneity. Sensitivity analyses were performed to explore the source of heterogeneity with detecting the influence of any single study on the prevalence or relative risk of outcomes. Statistical analysis was performed using STATA software (version 10; STATA, INC., College Station, TX, USA).

## Results

### Search results

**Fig. 1:**
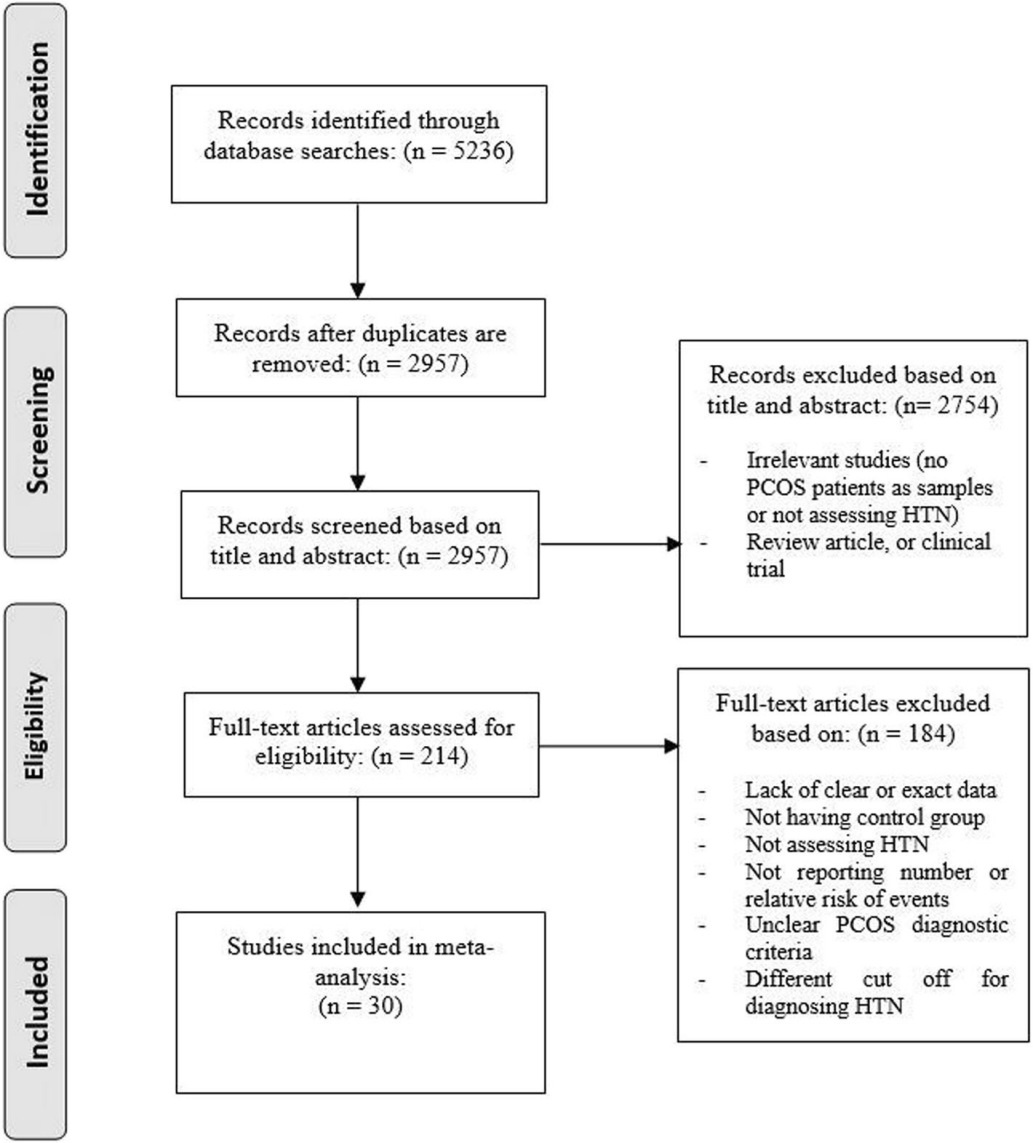
PRISMA flow diagram of search strategy and study selection

Figure 1 presents the search strategy and study selection. Of 5236 records retrieved through searching databases 30 studies including 17 population based [2, 3, 7, 19, 34–48] and 11 non-population based studies [4, 5, 10, 34, 49–56] were selected for the final analyses. Twenty-four studies assessed a population of reproductive age patients with PCOS [2, 3, 5, 7, 10, 34–38, 40, 41, 43–47, 49–51, 53–56], 4 studies- menopausal/aging women [19, 39, 52, 57], and two studies- both age groups of patients (reproductive and menopausal/aging women) [4, 48]. Table 1 shows characteristics of studies included. Details of quality assessment are presented as supplementary file 1.

**Table 1:** Characteristics of studies included in the meta-analysis

First author, year	Country	Study design	PCOS criteria	(a) BP measurement methods and standard conditions (b) Medication for hypertension (c) Diabetes status	PCOS group characteristics	Control group characteristics	Unadjusted RR (95% CI)	Quality assessment
Caldernon-Margalit et al. (2014) [2]	USA	Population based prospective cohort	NIH	(a) Not reported (b) Not reported (c) 7.3% diabetic in PCOS and 5.4% in control groups	*N* = 55 Age:45.4 (3.44); BMI: 29.3 (6.5)	*N* = 668 Age:45.40 (3.57); BMI: 29.90 (4.73)	1.03 (0.57, 1.85)	High
Chan et al. (2013) [34]	Australia	Case-control	Rotterdam	(a) Not reported (b) Not reported (c) 0% diabetic in PCOS vs. 3.7% in control groups	*N* = 109 Age = not reported; BMI = 31.6 (1.5)	*N* = 133 Age = not reported; BMI = 25.5 (1.40)	1.49 (0.64, 3.46)	Moderate
Chang et al. (2011) [35]	USA	Population based cross-sectional	Rotterdam	(a) Not reported (b) Not reported (c) 9% diabetic in PCOS and 8% in control groups	*N* = 144 Age = 40 (37–42)^*^; BMI = 31.7 (26.5–38.1)^*^	*N* = 170 Age = 42 (39–45)^*^; BMI = 28.7 (25.5–33.9)^*^	1.55 (1.03, 2.34)	High
Chang et al. (2016) [3]	USA	Population based cross-sectional	Rotterdam	(a) Not reported (b) Not reported (c) 9.4% diabetic in PCOS vs. 9.3% in control groups	*N* = 117 Age = 40.6^¥^; BMI = 31.06^¥^	*N* = 204 Age = 39.82^¥^; BMI = 31.43^¥^	1.51 (1.0005, 2.28)	Moderate
Dahlgren et al. (1992) [4]	Sweden	Prospective cohort study	Laparoscopic PCOS criteria	(a) Not reported (b) Not reported (c) 31.1% diabetic in PCOS vs. 4.8% in control groups	Group 1 (reproductive): *N* = 18 Age = 45.9 (2.5); BMI = no reported Group 22 (Menopause/aging): *N* = 15 Age = 55.1 (2.6); BMI = no reported	Group 1: *N* = 57 Age = 46 (2.2); BMI = No reported Group 2: *N* = 75 Age = 55.6 (3.1); BMI = no reported	RR for group 1: 7.9 (1.68, 37.16) RR for group 2: 3.8 (1.91, 7.55)	Moderate
Ding et al. (2018) [7]	Taiwan	Population based prospective cohort	ICD	(a) Note reported (b) Not reported (c) 2.94% diabetic in PCOS and 1.46% in control groups	*N* = 8048 Age = 28.11 (6.89); BMI = not reported	*N* = 32,192 Age = 28.11 (6.90); BMI = not reported	2.02 (1.73, 2.36)	High
Gateva et al. (2012) [5]	Bulgaria	Cross-sectional	Rotterdam	(a) Not reported (b) Not reported (c) Not reported	*N* = 81 Age = mean 25.98^€^; BMI = 36.21 (4.58)	*N* = 125 Age = 26.5 (5.47); BMI = 37.55 (5.95)	0.95 (0.58, 1.55)	Moderate
Glintborg et al. (2015) [36]	Denmark	Population based cohort	Rotterdam	(a) Not reported (b) 14% in PCOS and 6.7 in control groups (c) 2.24% in PCOS vs. 0.4% in control group	*N* = 20,416 Age = 29.3 (8.5); BMI = 27.3 (23.0–32.7) ^*^	*N* = 57,483 Age = 30.6 (9.6); BMI = 27.3 (23.0–32.7) ^*^	2.80 (2.44, 3.21)	Moderate
Haakova et al. (2003) [49]	Czech Republic	Case-control	NIH	(a) Not reported (b) Not reported (c) 13.64% in PCOS vs. 9.09% in control groups	*N* = 66 Age = 29.9 (2.97); BMI = 23.7 (4.27)	*N* = 66 Age = 29.8 (4.94); BMI = 23.2 (3.89)	1.0 (0.06, 15.55)	Moderate
Hart et al. (2015) [37]	Australia	Population based retrospective cohort	ICD	(a) Not reported (b) Not reported (c) 12.5% in PCOS vs. 3.8% in control group	*N* = 2560 Age = not reported; BMI = not reported	*N* = 25,660 Age = not reported; BMI = not reported	5.12 (4.05, 6.48)	High
Iftikhar et al. (2012) [50]	USA	Retrospective cohort	Rotterdam	(a) Not reported (b) Not reported (c) 0.7% in PCOS vs. 2.6% in control	*N* = 309 Age = not reported; BMI = 29.4 (7.77)	*N* = 343 Age = not reported; BMI = 28.3 (7.47)	1.22 (0.93, 1.61)	High
Lo et al. (2006) [38]	USA	Population based cross-sectional	ICD	(a) Not reported (b) Not reported (c) 9% in PCOS vs. 1.9% in control	*N* = 11,035 Age = 30.7 (7.2); BMI = not reported	*N* = 55,175 Age = 30.8 (7.5); BMI = not reported	2.49 (2.35, 2.64)	High
Lunde et al. (2007) [51]	Norway	Prospective cohort	Laparoscopic PCOS criteria	(a) Not reported (b) Not reported (c) 0.74% in PCOS vs. 0% in control	*N* = 131 Age = mean 24.7^€^; BMI = 24.7 (17.0–36.9) ^*^	*N* = 723 Age = not reported; BMI = not reported	1.1 (0.43, 2.3)	Moderate
Merz et al. (2016) [19]	USA	Population based prospective cohort	NIH	(a) Not reported (b) Not reported (c) 24% in PCOS vs. 32.2% in control	*N* = 25 Age = 62.6 (11.6); BMI = 28.7 (5.9)	*N* = 270 Age = 64.8 (9.8); BMI = 30.0 (6.7)	0.76 (0.50, 1.15)	High
Meun et. (2018) [39]	Netherlands	Population based prospective cohort	NIH	(a) Blood pressure was measured twice at the right brachial artery in sitting position with a random-zero sphygmomanometer (b) Not reported (c) 18.9% in PCOS vs. 7% in control	*N* = 106 Age = 69.57 (8.72); BMI = 27.92 (4.53)	*N* = 171 Age = 69.20 (8.60); BMI = 26.84 (3.83)	1.06 (0.89, 1.26)	High
Okoroh et al. (2015) [40]	USA	Population based cross-sectional	ICD	(a) Not reported (b) Not reported (c) 4.6% in PCOS vs. 1.9% in control	*N* = 125,268 Age = mean 33.4^€^; BMI = not reported	*N* = 250,536 Age = mean 33.4^€^; BMI = not reported	2.33 (2.28, 2.38)	High
Schmidt et al. (2011) [52]	Sweden	Prospective cohort	Rotterdam	(a) Blood pressure was measured (right arm, supine position) after 15 min rest. (b) Not reported (c) 22% in PCOS vs. 14% in control	*N* = 32 Age = not reported; BMI = not reported	*N* = 95 Age = not reported; BMI = not reported	1.67 (1.20, 2.33)	Moderate
Shi et al. (2014) [53]	China	Retrospective cohort	Rotterdam	(a) Blood pressure were determined from two blood pressure readings taken at 30-min intervals with the subject seated quietly. (b) Not reported (c) Not reported	*N* = 3396 Age = 30.49 (4.01); BMI = 24.97 (4.15)	*N* = 1891 Age = 30.73 (4.86); BMI = 22.69 (3.26)	1.61 (1.40, 1.85)	Moderate
Shroff et al. (2007) [10]	USA	Case-control	Rotterdam	(a) Mean systolic and diastolic blood pressure was assessed with two readings after 5 min of seated rest. (b) Not reported (c) 4.2% in PCOS vs. 0% in control group	*N* = 24 Age = 32 (6.5); BMI = 36 (5.4)	*N* = 24 Age = 36 (7.2); BMI = 35 (3.3)	1.5 (0.27, 8.25)	Moderate
Sirmans et al. (2014) [41]	USA	Population based cross-sectional	ICD	(a) Not reported (b) Not reported (c) 17.6% in PCOS vs. 4.7% in control group	*N* = 1689 Age = mean 25.24^€^; BMI = not reported	*N* = 5067 Age = mean 25.23; BMI = not reported	2.58 (2.45, 3.32)	Moderate
Vrbíková et al. (2003) [54]	Czech Republic	Cross-sectional	NIH	(a) Two blood pressure (BP) readings were obtained in sitting patients after a 10 min rest; the mean was determined from two values and was used for further analysis. (b) Not reported (c) 3.1% in PCOS vs. 0% in control group	*N* = 50 Age = 30.7 (4.2); BMI = 29.20 (7.10)	*N* = 335 Age = 29.9 (3.10); BMI = 24.10 (4.50)	2.73 (1.46, 5.11)	Moderate
Wild et al. (2000) [57]	UK	Population based retrospective cohort	Laparoscopic criteria	(a) Sitting blood pressures were measured twice in the right arm after participants had been resting for 5 min. Mean values for each individual were used in the analyses. (b) Not reported (c) 6.9% in PCOS vs. 3% in control group	*N* = 1060 Age = not reported; BMI = mean 25.9^€^	*N* = 319 Age = not reported; BMI = mean 26.6	1.2 (0.95, 1.52)	High
Bird et al. (2013) [43]	Canada	Population based cohort	ICD	(a) Not reported (b) 6.6 in PCOS vs. 7.1 in control (c) 20.3% in PCOS vs. 20.7% in control group	*N* = 43,506 Age = 28.7^€^; BMI = not reported	*N* = 43,506 Age = 28.9^€^; BMI = not reported	1.27 (1.22, 1.32)	Moderate
Li et al. (2013) [44]	China	Population based cross-sectional	Rotterdam	(a) Not reported (b) Not reported (c) Not reported	*N* = 833 Age = 29.1 (5.4); BMI = 22.2 (4.2)	*N* = 2732 Age = 32.3 (6.1); BMI = 22.2 (3.4)	1.004 (0.84, 1.20)	High
Ramezani Tehrani et al. (2015) [58]	Iran	Population based prospective cohort	NIH	(a) BP were measured twice on the right arm with the subject in a seated position with a standard mercury sphygmomanometer after the subject sat for 15 min; the mean of these 2 measurements was recorded (b) Not reported (c) Not reported	*N* = 85 Age = 29.8 (9.2); BMI = 27.2 (5.3)	*N* = 552 Age = 29.3 (9.0); BMI = 25.6 (5.0)	1.39 (0.60, 3.23)	High
Luque-Ramirez et al. (2007) [55]	Spain	Case-control	AES	(a) Ambulatory blood pressure monitoring (b) Not reported (c) Not reported	*N* = 36 Age = 24.2 (6.2); BMI = 29.3 (6.4)	*N* = 20 Age = 26.7 (6.8); BMI = 28.2 (6.9)	0.83 (0.41, 1.68)	Moderate
Ramezani Tehrani et al. (2011) [46]	Iran	Population based cross-sectional	Rotterdam	(a) Not reported (b) Not reported (c) Not reported	*N* = 136 Age = 32.4 (25.9–38.8)^*^; BMI = 25.9 (23.5–32.6)^*^	*N* = 423 Age = 36 (30–41.0) ^*^; BMI = 26.4 (23.1–29.4) ^*^	1.04 (0.29, 3.79)	High
Ramezani Tehrani et al. (2014) [47]	Iran	Population based cross-sectional	Rotterdam	(a) BP were measured twice on the right arm with the subject in a seated position with a standard mercury sphygmomanometer after the subject sat for 15 min; the mean of these 2 measurements was recorded (b) Not reported (c) Not reported	*N* = 85 Age = 29.02 (7.4)^*^; BMI = 26.5 (5.8)	*N* = 517 Age = 33.9 (7.6); BMI = 26.6 (5)	0.68 (0.09, 5.20)	High
Marchesan et al. (2019) [56]	Brazil	Cross-sectional	Rotterdam	(a) Blood pressure (measured after a 10-min rest, in the sitting position, with feet on the floor and the arm supported at heart level (b) Not reported (c) Not reported	*N* = 180 Age = 25 (21–29)^*^; BMI =32.52 (7.41)	*N* = 70 Age = 29 (26–34)^*^; BMI =28.71 (5.71)	2.29 (1.20, 4.37)	High
Behboudi-Gandevani et al. (2018) [48]	Iran	Population based cohort study	NIH	(a) BP were measured twice on the right arm with the subject in a seated position with a standard mercury sphygmomanometer after the subject sat for 15 min; the mean of these 2 measurements was recorded (b) Not reported (c) 1.1% in PCOS vs. 2.1% in control group	Group 1 (reproductive): *N* = 131 Age = 25.28 (7.69); BMI =25.89 (5.13) Group 2 (Menopause/aging): *N* = 28 Age = 46.19 (11.02); BMI =29.53 (4.51)	Group 1: *N* = 1046 Age = 26.57 (7.51); BMI = 24.89 (4.9) Group 2: *N* = 355 Age = 49.46 (6.26); BMI = 29.32 (4.57)	Group 1: 1.29 (0.89, 1.87) Group 2: 1.01 (0.61–1.68)	High

Abbreviations: *PCOS* Polycystic ovary syndrome, *N* Number, *RR* Relative risk, *CI* Confidence interval, *NIH* National Institutes of Health, *ICD* International Classification of Diseases

*Values represent median and interquartile

€ Values represent mean; standard deviation is not reported

¥ Values represent median; interquartile range is not reported

### Meta-analysis and meta-regression of outcomes

The review showed that the pooled prevalence of HTN in reproductive and menopausal/aging women with PCOS was higher than in the general population [(Pooled P: 0.15, 95% CI: 0.12–0.18 vs. Pooled P: 0.09, 95% CI: 0.08–0.10) and (Pooled P: 0.49, 95% CI: 0.28–0.70 vs. Pooled P: 0.40, 95% CI: 0.22–0.57), respectively]. The same results were obtained when only population-based studies were included in the meta-analysis [(Pooled P: 0.12, 95% CI: 0.08–0.15 vs. Pooled P: 0.08, 95% CI: 0.06–0.09) and (Pooled P: 0.60, 95% CI: 0.52–0.68 vs. Pooled P: 0.44, 95% CI: 0.40–0.48), respectively] (Table 2).

**Table 2:** Meta-analysis of studies included conducted on the prevalence of HTN

HTN	Number of observations	I^**2**^	^a^Publication bias	Pooled Prevalence (95%CI)
**All studies**				
Reproductive age	52	99	0.567	0.11 (0.10, 0.12)
Case	26	99	0.459	0.15 (0.12, 0.18)
Control	26	99	0.867	0.09 (0.08, 0.10)
Menopause/ aging	12	95	0.498	0.44 (0.32, 0.56)
Case	6	65	0.504	0.49 (0.28, 0.70)
Control	6	97	0.259	0.40 (0.22, 0.57)
**Population based studies**				
Reproductive age	28	99	0.768	0.09 (0.08, 0.11)
Case	14	99	0.343	0.12 (0.08, 0.15)
Control	14	99	0.218	0.08 (0.06, 0.09)
Menopause/ aging	4	94	0.250	0.50 (0.33, 0.68)
Case	2	–	0.317	0.60 (0.52, 0.68)
Control	2	–	0.317	0.44 (0.40, 0.48)
**Non-Population based studies**				
Reproductive age	24	93	0.768	0.16 (0.12, 0.19)
Case	12	94	0.987	0.20 (0.14, 0.26)
Control	12	94	0.800	0.12 (0.08, 0.16)
Menopause/ aging	8	93	0.328	0.41 (0.26, 0.55)
Case	4	97	0.250	0.47 (0.21, 0.73)
Control	4	95	0.243	0.35 (0.11, 0.59)

*I*^*2*^ I-squared

^a^ assessed by Begg’s test

**Fig. 2:**
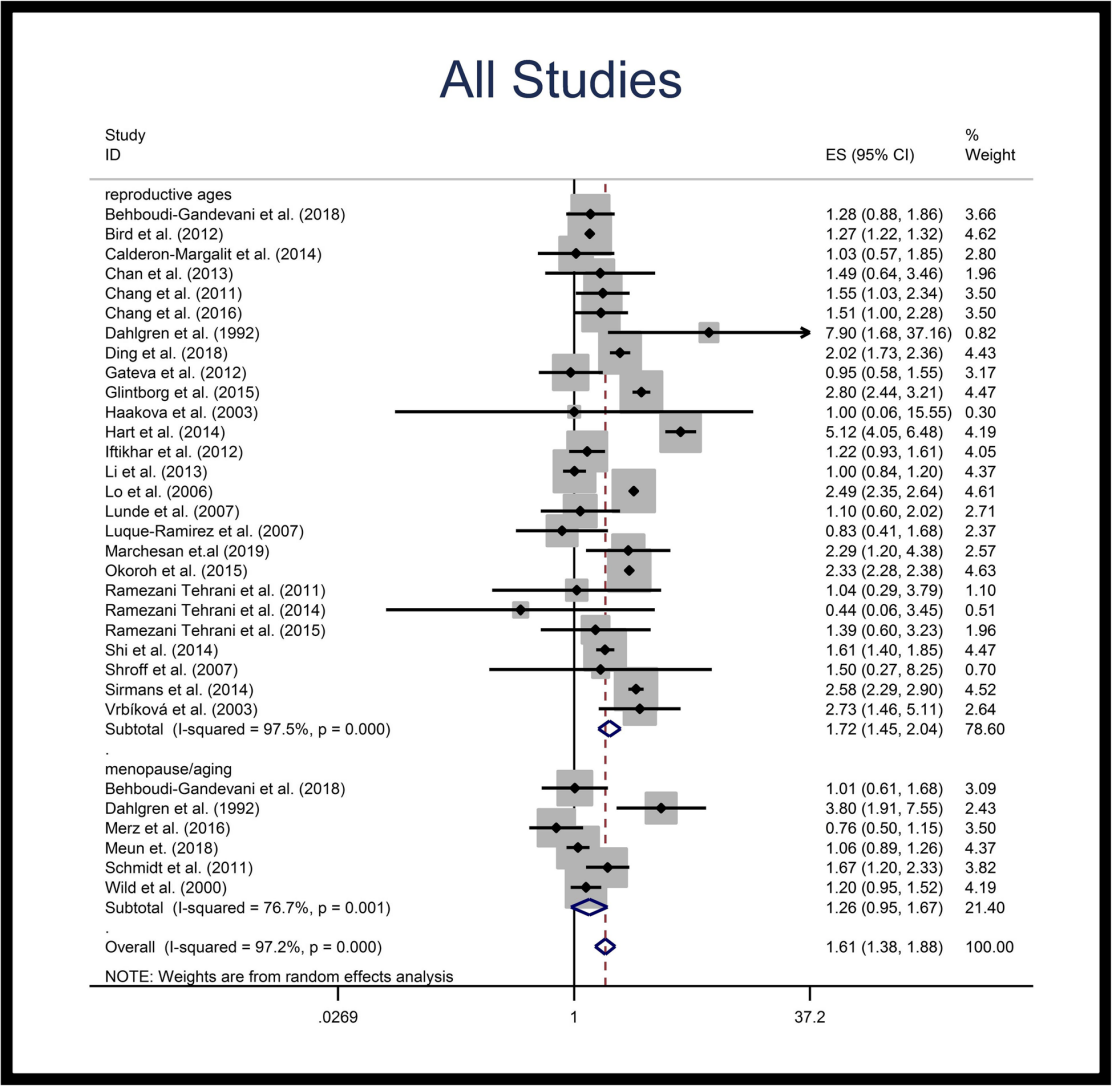
Forest plot of pooled relative risk of hypertension. Relative risk < 1 shows measures of in favor of PCOS (left side) and relative risk values > 1 are in favor of control population (right side)

**Fig. 3:**
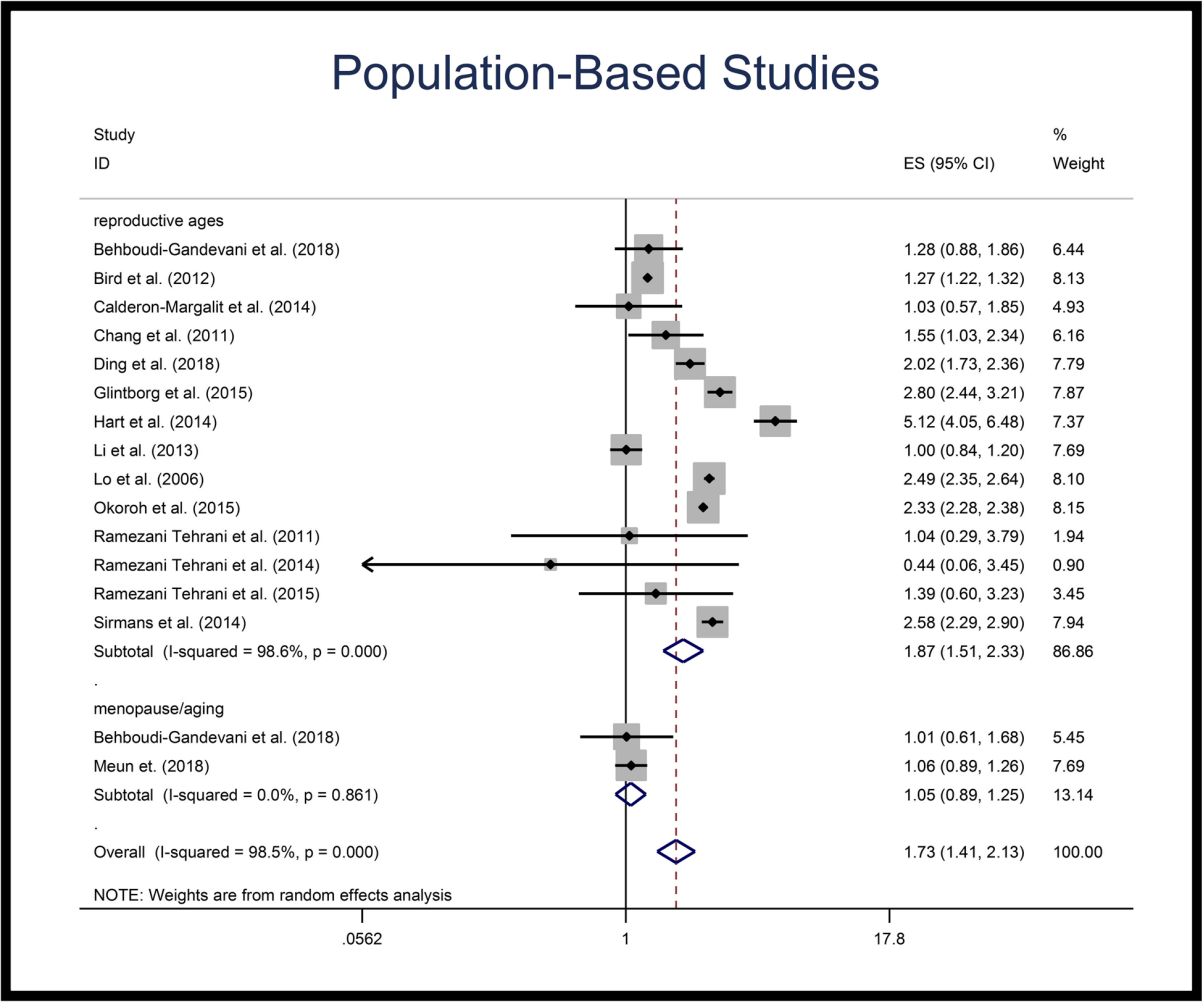
Forest plot of pooled relative risk of hypertension for population based studies. Relative risk < 1 shows measures of in favor of PCOS (left side) and relative risk values > 1 are in favor of control population (right side)

**Fig. 4:**
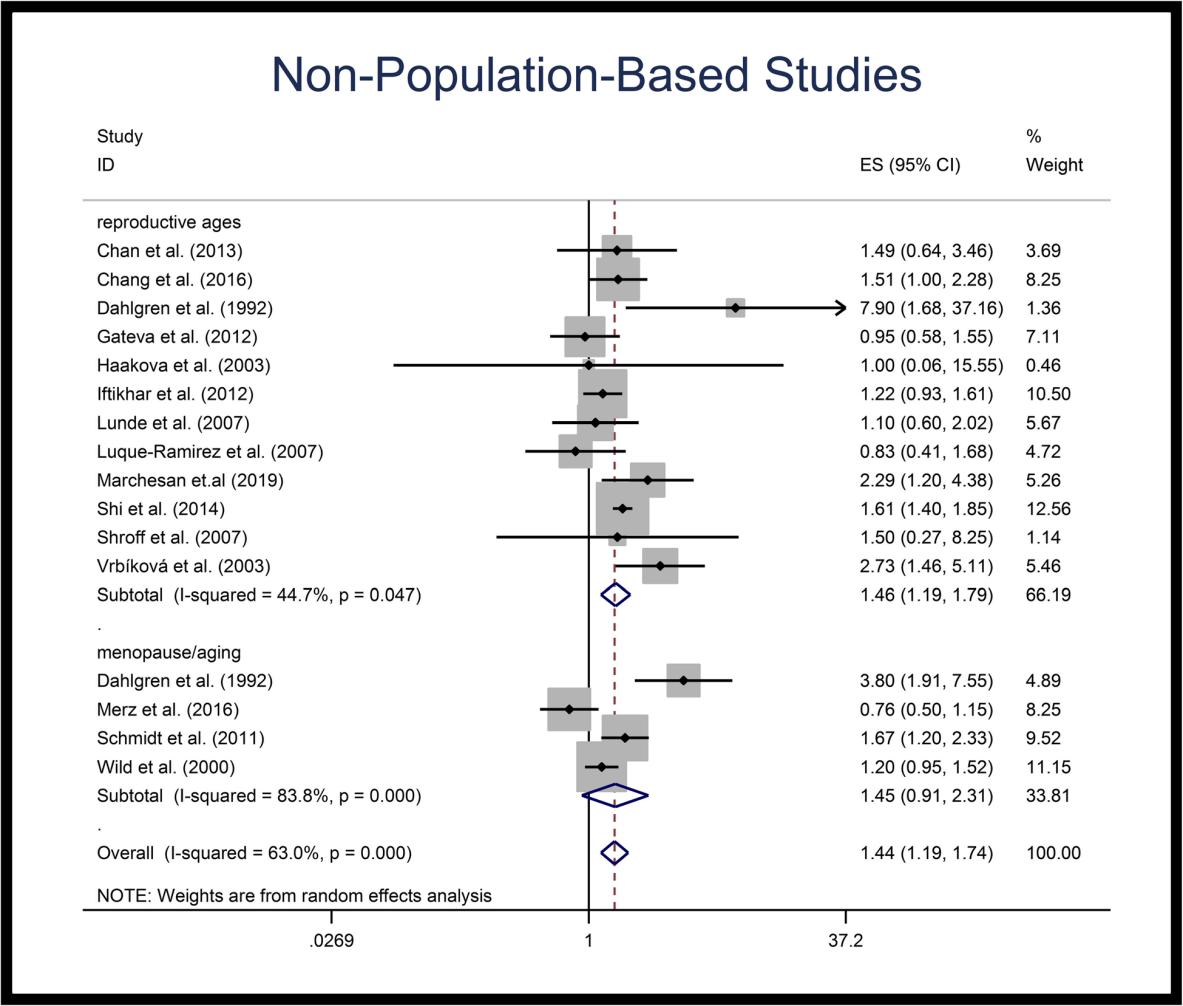
Forest plot of pooled relative risk of hypertension for non-population based studies. Relative risk < 1 shows measures of in favor of PCOS (left side) and relative risk values > 1 are in favor of control population (right side)

Figures 2, 3 and 4 illustrate the forest plots of pooled RRs of HTN in patients with PCOS, compared to control population. The RR of HTN in patients with PCOS was 1.60-fold (95% CI: 1.36–1.87) higher than in control population. Subgroup analysis based on the age groups revealed that the pooled RR of HTN in reproductive age PCOS patients was 1.70-fold (95% CI: 1.43–2.07) higher than in control populations of similar ages, but that the RR of HTN in menopausal/aging patients was not significantly different compared to the control population (Table 3, Fig. 2). Subgroup analysis of population-based studies revealed the same results; the pooled RR of HTN in reproductive age PCOS patients was 1.87-fold (95% CI: 1.51–2.33) higher than control population, while no significant difference was observed for menopausal/aging group (Table 3, Fig. 3). Figure 4 illustrates the forest plot of pooled RR of HTN for non-population based studies.

**Table 3:** Meta-analysis of studies included conducted on the relative risk (RR) of HTN

HTN	Number of study groups	I^**2**^%	^a^Publication bias	Pooled RR (95%CI)
**All studies**				
Reproductive	25	97	0.779	**1.70 (1.43, 2.07)**
Menopause/aging	6	85	0.497	1.26 (0.95, 1.67)
Total	21	97	0.586	**1.60 (1.36, 1.87)**
**Population based studies**				
Reproductive	14	99	0.213	**1.87 (1.51, 2.33)**
Menopause/aging	2	–	0.317	1.06 (0.89, 1.25)
Total	16	99	0.186	**1.73 (1.41, 2.13)**
**Non- Population based studies**				
Reproductive	11	47	0.675	**1.41 (1.14, 1.71)**
Menopause/aging	4	88	0.317	1.45 (0.91, 2.31)
Total	15	64	0.465	**1.40 (1.16, 1.71)**

*I*^*2*^ I-squared

^a^ assessed by Begg’s test

**Fig. 5:**
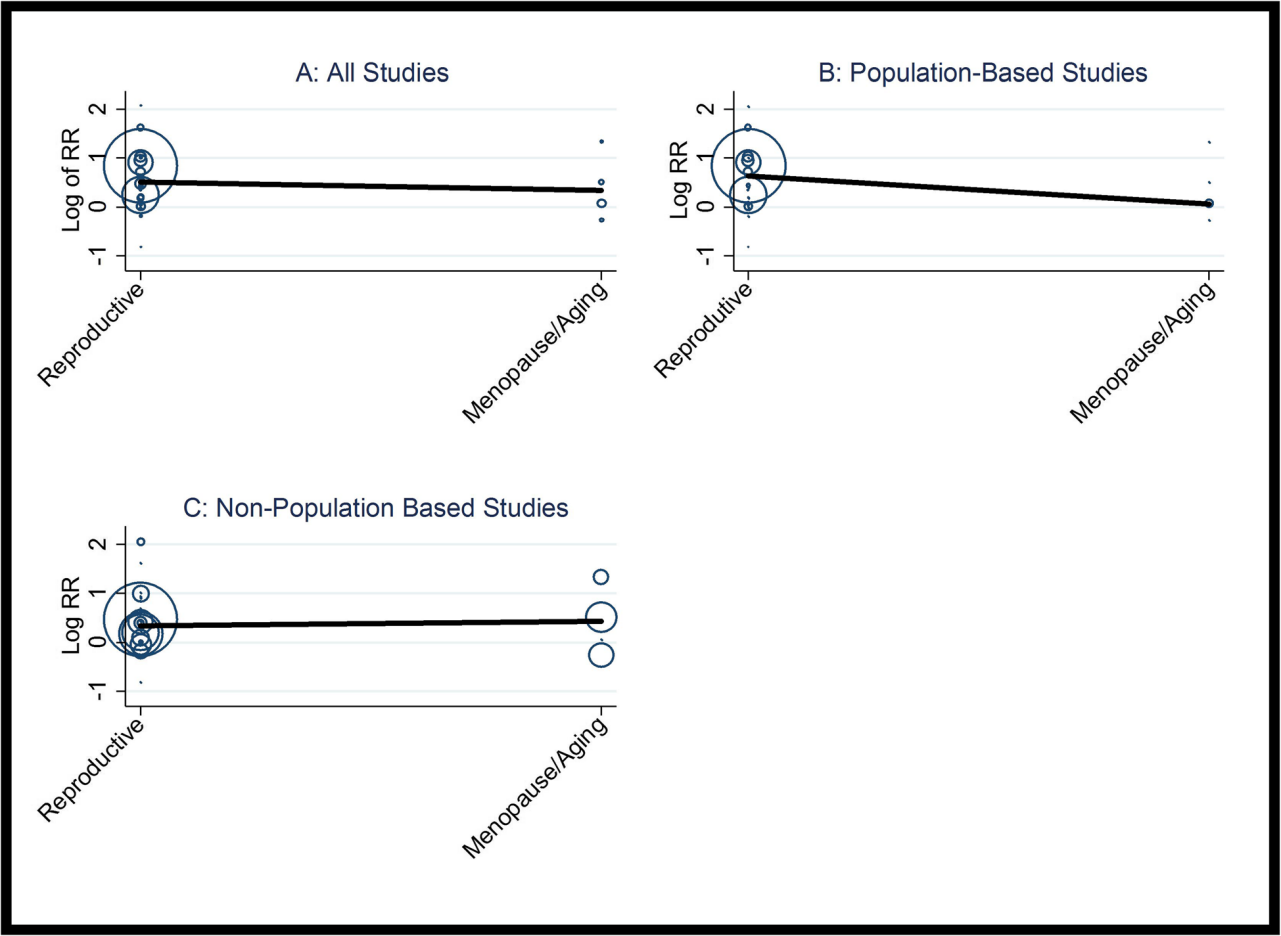
Bubble plot of association between relative risk of hypertension and age group (**a** total studies, **b** population-based studies, **c** non-population based studies). The solid black line represents the weighted regression line based on variance-weighted least squares. The circles indicate RRs in each study. The circle size is proportional to the precision of the RR. The vertical axis is on a log scale

Meta-regression analysis based on all studies, population-based and non-population-based studies is presented in Fig. 5. Meta-regression analysis of population-based studies showed that the RR of HTN in reproductive age PCOS patients was 1.76-fold (95% CI: 0.65–5.30) higher than those of HTN in menopausal/aging PCOS patients (*P* = 0.262). After adjusting the model with BMI and diabetes mellitus result did not significantly change (Table 4).

**Table 4:** Meta-regression results adjusted for BMI, and diabetes mellitus

Outcomes	Crud Regression Coefficient (95%CI)^a^	BMI-Adjusted Regression Coefficient(95%CI)	Diabetes-adjusted Regression Coefficient (95%CI)
All studies	0.86 (.47, 1.2),	0.80 (0.32, 1.8),	0.85 (0.38, 2.5),
	*P* = 0.225	*P* = 0.221	*P* = 0.129
Population-based studies	1.76 (0.65–5.30),	1.25(.81–1.93),	1.65 (0.72–1.81),
	P = 0.262	*P* = 0.125	*P* = 0.223
Non-population-based studies	0.71(.38, 1.3),	0.65 (0.22, 1.9),	0.71 (0.12, 2.1),
	*P* = 0.233	*P* = 0.325	*P* = 0.110

^a^Proportion of relative risk for reproductive vs menopause aging

There were significant heterogeneities in prevalence and RR of HTN among studies included in most subgroups of the study (Tables 2 and 3).

### Publication bias, risk of bias and sensitivity analysis

Egger’s test did not show any significant publication bias among studies included for HTN; therefore, trim and fill correction were not required (Tables 2 and 3).

Most cross-sectional and case-control studies had a low risk of bias in domains of assessment of exposure, development of outcome of interest in cases and controls, selection of cases, and selection of controls, and a high risk of bias in control of the prognostic variable. In the cohort studies, a low risk of bias selection of exposed and non-exposed cohorts, assessment of exposure, presence of outcome of interest at start of study, outcome assessment, and adequate follow up of cohorts were observed; however, we found a high risk of bias in control of prognostic variables and assessment of the presence or absence of prognostic factors (Supplementary File 1).

Sensitivity analysis suggested that the pooled RR and prevalence of hypertension were stable and excluding a single study did not change the significance of the pooled RR (Supplementary File 1). Since sensitivity analysis detected no significant heterogeneous study, the study type had no effect on the prevalence of hypertension. We also excluded the Rotterdam criteria and re-sun analyses; the results were analogues with the previous findings, indicating that the PCOS criteria had no significant effect on our results (supplementary File 1).

## Discussion

A large number of patients with PCOS, particularly those with hyperandrogenic phenotypes, present with several cardiometabolic risk factors that increase their chance for developing vascular abnormalities and hypertension [15, 59]. Androgen excess in PCOS may also directly influence the vascular properties of arterial walls and the expression of molecules involved in the atherogenic process [60]. However, contrasting results on the prevalence of hypertension in PCOS have been reported by some studies suggesting increased prevalence [3, 7, 35, 36, 38, 40, 41, 43], whereas others find no significant differences in general populations [2, 19, 39, 42, 44–47].

In this meta-analysis, we found that, prevalence of hypertension is higher in PCOS patients compared to control population. Limiting the included studies to population-based studies also showed that hypertension was more common in the PCOS than controls.

Because prevalence of HTN is increased in postmenopausal and aging women, we have separately assessed reproductive age PCOS patients and postmenopausal women who had PCOS during their reproductive period; results showed that, hypertension was more common only reproductive age PCOS women (OR 1.94, *p* = 0.01); this finding remained significant even after adjustment for BMI and diabetes mellitus.

Nevertheless, the data available suggest that the increased risk for HTN in PCOS patients ameliorates with aging [61], becoming normal in postmenopausal women who had PCOS during their reproductive age. Our current findings are parallel and in some way anticipate the data we observed in a recent meta-analysis that assesses the prevalence of cardiovascular events in PCOS subjects; in that study, cardiovascular events were also increased in young PCOS patients but normalized when postmenopausal PCOS women compared to control women of similar ages [62]. Because HTN is one of the main cardiovascular risks, it is probable that normalization of the prevalence of HTN with age plays a main role in the normalization of cardiovascular events.

Mechanisms determining the reduction of the risk of HTN by ageing are unclear, although the progressive decrease of androgens during adult reproductive ages may play a main role; a progressive decrease of serum testosterone in both general populations and in PCOS women is observed many years before the occurrence of menopause [63]. A follow-up study showed that approximately 50% of PCOS patients improve during late reproductive age because of ovarian and adrenal aging that leads to decreasing androgen levels, which can result in a progressive decrease in cardiovascular risk factors [64]. Other longitudinal studies demonstrated that some cardiovascular risk factors in women with PCOS may be progressively decreased with aging [45, 59] .

The main limitation of this meta-analysis is the small number of studies assessing HTN in postmenopausal PCOS women [4, 19, 39, 52]. In addition, some studies did not report a crude relative risk or the exact number of HTN cases, nor did have a risk of bias in the control of confounders. Although the risk of bias in these studies assessing postmenopausal women was low with no differences in RR for HTN compared to young patients. For this study, we used data of hypertension based on the previous JNC7 criteria (SBP ≥140 mmHg or DBP ≥90 mmHg) [56], moreover most studies did not report details of the BP measurement methods and standard conditions, which could affect the accuracy and validity of results; hence, caution should be considered to interpret findings. Finally, the possible influence of body weight and obesity on reported results could not be assessed because of the paucity of related data.

## Conclusions

Increasing risk for hypertension in PCOS women compared to controls is observed only in reproductive age but not in menopausal women with history of PCOS during reproductive period. After menopause, having a history of PCOS may not be as an important risk factor for developing HTN.

## Supplementary information

**Additional file 1: Table S1.** Quality assessment of included studies using the Newcastle–Ottawa Quality Assessment Scale for cross-sectional studies. **Table S2.** Quality assessment of included studies using the Newcastle–Ottawa Quality Assessment Scale for cohort studies. **Table S3.** Quality assessment of included studies using the Newcastle–Ottawa Quality Assessment Scale for case-control studies. **Figure S1.** Risk of bias in cross-sectional and case- control studies. **Figure S2.** Risk of bias in cohort studies. **Figure S3.** Sensitivity analysis for RR in reproductive age group for all studies. **Table S4.** sensitivity analysis for RR in reproductive age group for all studies. **Figure S4.** Sensitivity analysis for RR in menopause aging group for all studies. **Table S5.** Sensitivity analysis for RR in menopause aging group for all studies. **Figure S5.** Sensitivity analysis for Prevalence in patients with PCOS of reproductive ages. **Table S6.** Sensitivity analysis for Prevalence in patients with PCOS in reproductive ages. **Figure S6.** Sensitivity analysis for Prevalence in patients with PCOS in menopause aging group. **Table S7.** Sensitivity analysis for Prevalence in patients with PCOS in menopause aging group. **Figure S7.** Sensitivity analysis for Prevalence in healthy controls of reproductive ages. **Table S8.** Sensitivity analysis for Prevalence in healthy control of reproductive ages. **Figure S8.** Sensitivity analysis for Prevalence in healthy control of menopause aging group. **Table S9.** Sensitivity analysis for Prevalence in healthy control of menopause aging group. **Figure S9.** The result of sensitivity analysis for all age subgroups. **Figure S10.** The result of sensitivity analysis for reproductive age subgroup. **Figure S11.** The result of sensitivity analysis for menopause/aging subgroup. **Figure S12.** Forest plot of pooled relative risk of HTN for all studies except those with Rotterdam criteria. **Figure S13.** Forest plot of pooled relative risk of HTN for all population based studies except those with Rotterdam criteria. **Figure S14.** Forest plot of pooled relative risk of HTN for all non-population based studies except those with Rotterdam criteria.

## Data Availability

The current study was based on results of relevant published studies.

## Abbreviations

CV: Cardiovascular
DBP: Diastolic blood pressure
HTN: Hypertension
P: Prevalence
PCOS: Polycystic ovary syndrome
PRISMA: Preferred Reporting Items for Systematic Reviews and Meta-Analyses
RR: Relative risk
SBP: Systolic blood pressure

## References

[1] Goodarzi MO, Dumesic DA, Chazenbalk G, Azziz R (2011). Polycystic ovary syndrome: etiology, pathogenesis and diagnosis. Nat Rev Endocrinol.

[2] Calderon-Margalit R, Siscovick D, Merkin SS, Wang E, Daviglus ML, Schreiner PJ (2014). Prospective Association of Polycystic Ovary Syndrome with coronary artery calcification and carotid-intima-media thickness significance: the coronary artery risk development in young adults Women’s study. Arterioscler Thromb Vasc Biol.

[3] Chang AY, Oshiro J, Ayers C, Auchus RJ (2016). Influence of race/ethnicity on cardiovascular risk factors in polycystic ovary syndrome, the Dallas heart study. Clin Endocrinol (Oxf).

[4] Dahlgren E, Janson P, Johansson S, Lapidus L, Oden A (1992). Polycystic ovary syndrome and risk for myocardial infarction: evaluated from a risk factor model based on a prospective population study of women. Acta Obstet Gynecol Scand.

[5] Gateva A, Kamenov Z (2012). Cardiovascular risk factors in Bulgarian patients with polycystic ovary syndrome and/or obesity. Obstet Gynecol Int.

[6] Hillman JK, Johnson LN, Limaye M, Feldman RA, Sammel M, Dokras A (2014). Black women with polycystic ovary syndrome (PCOS) have increased risk for metabolic syndrome and cardiovascular disease compared with white women with PCOS. Fertil Steril.

[7] Ding D-C, Tsai I-J, Wang J-H, Lin S-Z, Sung F-C (2018). Coronary artery disease risk in young women with polycystic ovary syndrome. Oncotarget.

[8] Carmina E, Lobo RA (1999). Polycystic ovary syndrome (PCOS): arguably the most common endocrinopathy is associated with significant morbidity in women. J Clin Endocrinol Metabol.

[9] Daan NM, Louwers YV, Koster MP, Eijkemans MJ, de Rijke YB, Lentjes EW (2014). Cardiovascular and metabolic profiles amongst different polycystic ovary syndrome phenotypes: who is really at risk?. Fertil Steril.

[10] Shroff R, Kerchner A, Maifeld M, Van Beek EJ, Jagasia D, Dokras A (2007). Young obese women with polycystic ovary syndrome have evidence of early coronary atherosclerosis. J Clin Endocrinol Metabol.

[11] Christian RC, Dumesic DA, Behrenbeck T, Oberg AL, Sheedy PF, Fitzpatrick LA (2003). Prevalence and predictors of coronary artery calcification in women with polycystic ovary syndrome. J Clin Endocrinol Metabol.

[12] Tarkun I, Arslan BC, Canturk Z, Turemen E, Şahı̇n T, Duman C (2004). Endothelial dysfunction in young women with polycystic ovary syndrome: relationship with insulin resistance and low-grade chronic inflammation. J Clin Endocrinol Metabol.

[13] Talbott E, Clerici A, Berga SL, Kuller L, Guzick D, Detre K (1998). Adverse lipid and coronary heart disease risk profiles in young women with polycystic ovary syndrome: results of a case-control study. J Clin Epidemiol.

[14] Cibula D, Cifkova R, Fanta M, Poledne R, Zivny J, Skibova J (2000). Increased risk of non-insulin dependent diabetes mellitus, arterial hypertension and coronary artery disease in perimenopausal women with a history of the polycystic ovary syndrome. Hum Reprod.

[15] Joham AE, Boyle JA, Zoungas S, Teede HJ (2014). Hypertension in reproductive-aged women with polycystic ovary syndrome and association with obesity. Am J Hypertens.

[16] Holte J, Gennarelli G, Berne C, Bergh T, Lithell H (1996). Elevated ambulatory day-time blood pressure in women with polycystic ovary syndrome: a sign of a pre-hypertensive state?. Hum Reprod.

[17] Zimmermann S, Phillips RA, Dunaif A, Finegood DT, Wilkenfeld C, Ardeljan M (1992). Polycystic ovary syndrome: lack of hypertension despite profound insulin resistance. J Clin Endocrinol Metabol.

[18] Meyer C, McGrath B, Teede H (2005). Overweight women with polycystic ovary syndrome have evidence of subclinical cardiovascular disease. J Clin Endocrinol Metabol.

[19] Merz CNB, Shaw LJ, Azziz R, Stanczyk FZ, Sopko G, Braunstein GD (2016). Cardiovascular disease and 10-year mortality in postmenopausal women with clinical features of polycystic ovary syndrome. J Womens Health.

[20] Moher D, Liberati A, Tetzlaff J, Altman DG, Group P (2009). Preferred reporting items for systematic reviews and meta-analyses: the PRISMA statement. PLoS Med.

[21] Higgins JP, Green S. Cochrane handbook for systematic reviews of interventions, vol. 4. New York: Wiley; 2011.

[22] Carmina E (2004). Diagnosis of polycystic ovary syndrome: from NIH criteria to ESHRE-ASRM guidelines. Minerva Ginecol.

[23] Azziz R (2006). Diagnosis of polycystic ovarian syndrome: the Rotterdam criteria are premature. J Clin Endocrinol Metabol.

[24] Azziz R, Carmina E, Dewailly D, Diamanti-Kandarakis E, Escobar-Morreale HF, Futterweit W (2006). Criteria for defining polycystic ovary syndrome as a predominantly hyperandrogenic syndrome: an androgen excess society guideline. J Clin Endocrinol Metabol.

[25] Kandil M, Selim M (2005). Hormonal and sonographic assessment of ovarian reserve before and after laparoscopic ovarian drilling in polycystic ovary syndrome. BJOG.

[26] Escobar-Morreale HF (2018). Polycystic ovary syndrome: definition, aetiology, diagnosis and treatment. Nat Rev Endocrinol.

[27] Stang A (2010). Critical evaluation of the Newcastle-Ottawa scale for the assessment of the quality of nonrandomized studies in meta-analyses. Eur J Epidemiol.

[28] Higgins JP, Altman DG, Gøtzsche PC, Jüni P, Moher D, Oxman AD (2011). The Cochrane Collaboration’s tool for assessing risk of bias in randomised trials. Bmj.

[29] Chobanian AV (2003). National heart, lung, and blood institute joint national committee on prevention, detection, evaluation, and treatment of high blood pressure; national high blood pressure education program coordinating committee: the seventh report of the joint national committee on prevention, detection, evaluation, and treatment of high blood pressure: the JNC 7 report. Jama.

[30] Begg CB, Mazumdar M (1994). Operating characteristics of a rank correlation test for publication bias. Biometrics.

[31] Duval S, Tweedie R (2000). Trim and fill: a simple funnel-plot–based method of testing and adjusting for publication bias in meta-analysis. Biometrics.

[32] Nyaga VN, Arbyn M, Aerts M (2014). Metaprop: a Stata command to perform meta-analysis of binomial data. Arch Publ Health.

[33] L'ABBÉ KA, Detsky AS, O'rourke K (1987). Meta-analysis in clinical research. Ann Intern Med.

[34] Chan WPA, Ngo DT, Sverdlov AL, Rajendran S, Stafford I, Heresztyn T (2013). Premature aging of cardiovascular/platelet function in polycystic ovarian syndrome. Am J Med.

[35] Chang AY, Ayers C, Minhajuddin A, Jain T, Nurenberg P, De Lemos JA (2011). Polycystic ovarian syndrome and subclinical atherosclerosis among women of reproductive age in the Dallas heart study. Clin Endocrinol (Oxf).

[36] Glintborg D, Rubin KH, Nybo M, Abrahamsen B, Andersen M (2015). Morbidity and medicine prescriptions in a nationwide Danish population of patients diagnosed with polycystic ovary syndrome. Eur J Endocrinol.

[37] Hart R, Doherty DA (2015). The potential implications of a PCOS diagnosis on a woman’s long-term health using data linkage. J Clin Endocrinol Metabol.

[38] Lo JC, Feigenbaum SL, Yang J, Pressman AR, Selby JV, Go AS (2006). Epidemiology and adverse cardiovascular risk profile of diagnosed polycystic ovary syndrome. J Clin Endocrinol Metabol.

[39] Meun C, Franco OH, Dhana K, Jaspers L, Muka T, Louwers Y (2018). High androgens in postmenopausal women and the risk for atherosclerosis and cardiovascular disease: the Rotterdam study. J Clin Endocrinol Metabol.

[40] Okoroh EM, Boulet SL, George MG, Hooper WC (2015). Assessing the intersection of cardiovascular disease, venous thromboembolism, and polycystic ovary syndrome. Thromb Res.

[41] Sirmans SM, Parish RC, Blake S, Wang X (2014). Epidemiology and comorbidities of polycystic ovary syndrome in an indigent population. J Invest Med.

[42] Wild S, Pierpoint T, McKeigue P, Jacobs H (2000). Cardiovascular disease in women with polycystic ovary syndrome at long-term follow-up: a retrospective cohort study. Clin Endocrinol (Oxf).

[43] Bird ST, Hartzema AG, Brophy JM, Etminan M, Delaney JA (2013). Risk of venous thromboembolism in women with polycystic ovary syndrome: a population-based matched cohort analysis. Can Med Assoc J.

[44] Li R, Zhang Q, Yang D, Li S, Lu S, Wu X (2013). Prevalence of polycystic ovary syndrome in women in China: a large community-based study. Hum Reprod.

[45] Tehrani FR, Montazeri SA, Hosseinpanah F, Cheraghi L, Erfani H, Tohidi M (2015). Trend of cardio-metabolic risk factors in polycystic ovary syndrome: a population-based prospective cohort study. PLoS One.

[46] Tehrani FR, Simbar M, Tohidi M, Hosseinpanah F, Azizi F. The prevalence of polycystic ovary syndrome in a community sample of Iranian population: Iranian PCOS prevalence study. Reprod Biol Endocrinol. 2011;9:39. 10.1186/1477-7827-9-39 PMC3070632 21435276

[47] Tehrani FR, Rashidi H, Khomami MB, Tohidi M, Azizi F. The prevalence of metabolic disorders in various phenotypes of polycystic ovary syndrome: a community based study in southwest of Iran. Reprod Biol Endocrinol. 2014;12:89. 10.1186/1477-7827-12-89 PMC4180586 25224635

[48] Behboudi-Gandevani S, Tehrani FR, Hosseinpanah F, Khalili D, Cheraghi L, Kazemijaliseh H (2018). Cardiometabolic risks in polycystic ovary syndrome: long-term population-based follow-up study. Fertil Steril.

[49] Haakova L, Cibula D, Rezabek K, Hill M, Fanta M, Zivny J (2003). Pregnancy outcome in women with PCOS and in controls matched by age and weight. Hum Reprod.

[50] Iftikhar S, Collazo-Clavell M, Roger VL, Sauver JS, Brown R, Cha S (2012). Risk of cardiovascular events in patients with polycystic ovary syndrome. Neth J Med.

[51] Lunde O, Tanbo T (2007). Polycystic ovary syndrome: a follow-up study on diabetes mellitus, cardiovascular disease and malignancy 15–25 years after ovarian wedge resection. Gynecol Endocrinol.

[52] Schmidt J, Landin-Wilhelmsen K, Brännström M, Dahlgren E (2011). Cardiovascular disease and risk factors in PCOS women of postmenopausal age: a 21-year controlled follow-up study. J Clin Endocrinol Metabol.

[53] Shi Y, Cui Y, Sun X, Ma G, Ma Z, Gao Q (2014). Hypertension in women with polycystic ovary syndrome: prevalence and associated cardiovascular risk factors. Eur J Obstet Gynecol Reprod Biol.

[54] Vrbikova J, Cifkova R, Jirkovska A, Lanska V, Platilova H, Zamrazil V (2003). Cardiovascular risk factors in young Czech females with polycystic ovary syndrome. Hum Reprod.

[55] Luque-Ramírez M, Mendieta-Azcona C, Alvarez-Blasco F, Escobar-Morreale HF (2007). Androgen excess is associated with the increased carotid intima-media thickness observed in young women with polycystic ovary syndrome. Hum Reprod.

[56] Marchesan LB, Spritzer PM (2019). ACC/AHA 2017 definition of high blood pressure: implications for women with polycystic ovary syndrome. Fertil Steril.

[57] Wild SH (2000). Mortality and morbidity from coronary heart disease, diabetes and hypertension in women with polycystic ovary syndrome at long-term follow-up.

[58] Ramezani Tehrani F, Montazeri SA, Hosseinpanah F, Cheraghi L, Erfani H, Tohidi M, Azizi F (2015). Trend of Cardio-Metabolic Risk Factors in Polycystic Ovary Syndrome: A Population-Based Prospective Cohort Study. PLoS One.

[59] Jaliseh HK, Tehrani FR, Behboudi-Gandevani S, Hosseinpanah F, Khalili D, Cheraghi L (2017). Polycystic ovary syndrome is a risk factor for diabetes and prediabetes in middle-aged but not elderly women: a long-term population-based follow-up study. Fertil Steril.

[60] Christakou CD, Diamanti-Kandarakis E (2008). Role of androgen excess on metabolic aberrations and cardiovascular risk in women with polycystic ovary syndrome. Womens Health.

[61] Azziz R. Does the risk of diabetes and heart disease in women with polycystic ovary syndrome lessen with age? Elsevier; 2017. 10.1016/j.fertnstert.2017.09.034 29202973

[62] Ramezani Tehrani F, Amiri M, Behboudi-Gandevani S, Bidhendi-Yarandi R, Carmina E. Cardiovascular events among reproductive and menopausal age women with polycystic ovary syndrome: a systematic review and meta-analysis. Gynecol Endocrinol. 2020;36(1):12–23. 10.1080/09513590.2019.1650337 31385729

[63] Carmina E (2009). Cardiovascular risk and events in polycystic ovary syndrome. Climacteric.

[64] Carmina E, Campagna A, Lobo R (2013). Emergence of ovulatory cycles with aging in women with polycystic ovary syndrome (PCOS) alters the trajectory of cardiovascular and metabolic risk factors. Hum Reprod.

