# Supplementary material for: Risk of hypertension in women with polycystic ovary syndrome: a systematic review, meta-analysis and meta-regression
Source: Reprod Biol Endocrinol. 2020 Mar 17;18:23. doi: 10.1186/s12958-020-00576-1 (PMC7076940; doi:10.1186/s12958-020-00576-1)
Supplement: Supplementary file 1 — Additional file 1: Table S1. Quality assessment of included studies using the Newcastle–Ottawa Quality Assessment Scale for cross-sectional studies. Table S2. Quality assessment of included studies using the Newcastle–Ottawa Quality Assessment Scale for cohort studies. Table S3. Quality assessment of included studies using the Newcastle–Ottawa Quality Assessment Scale for case-control studies. Figure S1. Risk of bias in cross-sectional and case- control studies. Figure S2. Risk of bias in cohort studies. Figure S3. Sensitivity analysis for RR in reproductive age group for all studies. Table S4. sensitivity analysis for RR in reproductive age group for all studies. Figure S4. Sensitivity analysis for RR in menopause aging group for all studies. Table S5. Sensitivity analysis for RR in menopause aging group for all studies. Figure S5. Sensitivity analysis for Prevalence in patients with PCOS of reproductive ages. Table S6. Sensitivity analysis for Prevalence in patients with PCOS in reproductive ages. Figure S6. Sensitivity analysis for Prevalence in patients with PCOS in menopause aging group. Table S7. Sensitivity analysis for Prevalence in patients with PCOS in menopause aging group. Figure S7. Sensitivity analysis for Prevalence in healthy controls of reproductive ages. Table S8. Sensitivity analysis for Prevalence in healthy control of reproductive ages. Figure S8. Sensitivity analysis for Prevalence in healthy control of menopause aging group. Table S9. Sensitivity analysis for Prevalence in healthy control of menopause aging group. Figure S9. The result of sensitivity analysis for all age subgroups. Figure S10. The result of sensitivity analysis for reproductive age subgroup. Figure S11. The result of sensitivity analysis for menopause/aging subgroup. Figure S12. Forest plot of pooled relative risk of HTN for all studies except those with Rotterdam criteria. Figure S13. Forest plot of pooled relative risk of HTN for all population based studies except those wit [file 12958_2020_576_MOESM1_ESM.docx]

**Supplementary file 1**

**Quality assessment**

| Author | SELECTION | | | | COMPARABILITY | Outcome | | Total scores |
| --- | --- | --- | --- | --- | --- | --- | --- | --- |
|  | Representativeness of the samples | Sample size | Non-responders | Ascertainment of the exposure (risk factor) | A: study controls for age and/or BMI  B: control for any additional factor | Assessment of the outcome  a) Independent blind assessment. **  b) Record linkage. **  c) Self report. * | Statistical test |  |
| Chang, 2011 | * | * | - | * | ** | ** | * | 8 |
| Lo, 2006 | * | - | - | * | ** | ** | * | 7 |
| Okoroh, 2015 | * | * | * | * | * | ** | * | 8 |
| Sirmans, 2014 | * | * | - | * | - | ** | * | 6 |
| Chang, 2016 | - | - | - | * | * | ** | * | 5 |
| Vrbíková, 2003 | - | - | * | * | - | ** | * | 5 |
| Gateva, 2012 | - | - | - | * | - | ** | * | 4 |
| Li, 2013 | * | * | * | * | - | ** | * | 7 |
| Ramezani Tehrani, 2011 | * | * | * | * | - | ** | * | 7 |
| Ramezani Tehrani, 2014 | * | * | * | * | * | ** | * | 8 |
| Marchesan, 2019 | * | - | * | * | * | ** | * | 7 |

**Table S1.** Quality assessment of included studies using the Newcastle–Ottawa Quality Assessment Scale for cross-sectional studies.

|  | SELECTION | | | | COMPARABILITY | Outcome | | | Total scores |
| --- | --- | --- | --- | --- | --- | --- | --- | --- | --- |
| Author | Representativeness of the exposed cohort | Selection of the non-exposed cohort | Ascertainment of exposure | No outcome of interest at start of study | A: Study controls for age and/or BMI  B: Study controls for other confounders | A: doctor’s diagnosis OR objective measurements  B: parent/self-reported doctor’s diagnosis OR use of medication | follow-up long enough for outcomes ( at least 10 year) | Adequacy of follow up of cohorts |  |
| Calderon-Margalit, 2014 | * | * | * | * | ** | * | * | * | 9 |
| Ding, 2018 | * | * | * | * | ** | * | * | * | 9 |
| Glintborg, 2015 | * | * | * | * | - | * | - | - | 5 |
| Hart, 2014 (98) | * | * | * | * | - | * | * | * | 7 |
| Merz, 2016 | * | * | * | * | - | * | * | * | 7 |
| Meun, 2018 | * | * | * | * | ** | * | * | * | 9 |
| Wild, 2000 | * | * | * | * | - | * | * | * | 7 |
| Dahlgren, 1992 | * | - | * | * | - | * | * | * | 6 |
| Iftikhar, 2012 | * | - | * | * | ** | * | * | * | 8 |
| Lunde, 2007 | * | - | * | * | - | * | * | * | 6 |
| Schmidt, 2011 | * | - | * | * | - | * | * | * | 6 |
| Shi, 2014 | * | * | * | * | - | * | - | - | 5 |
| Bird, 2012 | * | * | * | * | - | * | - | - | 5 |
| Ramezani Tehrani, 2015 | * | * | * | * | ** | * | * | * | 9 |
| Behboudi Gandevani, 2018 | * | * | * | * | ** | * | * | * | 9 |

**Table S2.** Quality assessment of included studies using the Newcastle–Ottawa Quality Assessment Scale for cohort studies.

**Table S3.** Quality assessment of included studies using the Newcastle–Ottawa Quality Assessment Scale for case-control studies.

| First author, year  (reference) | SELECTION | | | | COMPARABILITY | EXPOSURE | | | Total scores |
| --- | --- | --- | --- | --- | --- | --- | --- | --- | --- |
|  | adequate case definition | Representativeness of the cases | Community selection of controls | no history of disease among controls | A: study controls for age and/or BMI  B: study control for any additional factors | A: secure record for clinical outcome  B: structured interview where blind to case/control status | Same method of ascertainment for cases and controls | Same Non-response rate for cases and controls |  |
| Chan, 2013 | * | * | - | * | - | * | * | - | 5 |
| Haakova, 2003 | * | * | - | * | - | * | * | - | 5 |
| Shroff , 2007 | * | * | - | * | - | * | * | * | 6 |
| Luque-Ramirez, 2007 | * | * | - | * | - | * | * | * | 6 |

**Risk of bias**

**Figure S1.** Risk of bias in cross-sectional and case- control studies.

| Study design | First author, date | Bias in assessment of exposure | Bias in development of outcome of interest in case and controls | Bias in selection of cases | Bias in selection of controls | Bias in control of prognostic variable (without case and control matching or adjustment in statistical methods ) |
| --- | --- | --- | --- | --- | --- | --- |
| cross-sectional | **Chang, 2011** |  |  |  |  |  |
|  | **Lo, 2006** |  |  |  |  |  |
|  | **Okoroh, 2015** |  |  |  |  |  |
|  | **Sirmans, 2014** |  |  |  |  |  |
|  | **Chang, 2016** |  |  |  |  |  |
|  | **Vrbíková, 2003** |  |  |  |  |  |
|  | **Li, 2013** |  |  |  |  |  |
|  | **Gateva, 2012** |  |  |  |  |  |
|  | **Ramezani Tehrani, 2011** |  |  |  |  |  |
|  | **Ramezani Tehrani, 2014** |  |  |  |  |  |
|  | **Marchesan, 2019** |  |  |  |  |  |
| case-control | **Chan, 2013** |  |  |  |  |  |
|  | **Haakova, 2003** |  |  |  |  |  |
|  | **Shroff , 2007** |  |  |  |  |  |
|  | **Luque-Ramirez, 2007** |  |  |  |  |  |
| Definitely No (low risk of bias) probably no  Definitely yes (high risk of bias) probably Yes | | | | | | |

**A.**

**B.**

**Figure S2.** Risk of bias in cohort studies.

**A.**

| Author, date | Bias in selection of exposed and non‐exposed cohorts | Bias in assessment of exposure | Bias in present of outcome of interest at start of study | Bias in control of prognostic variables (with matching or adjusting) | Bias in in the assessment of the presence or absence of prognostic factors | Bias in in the assessment of outcome | Bias in adequacy about follow up of cohorts |
| --- | --- | --- | --- | --- | --- | --- | --- |
| Ding, 2018 |  |  |  |  |  |  |  |
| Calderon-Margalit, 2014 |  |  |  |  |  |  |  |
| Glintborg, 2015 |  |  |  |  |  |  |  |
| Hart, 2014 |  |  |  |  |  |  |  |
| Merz, 2016 |  |  |  |  |  |  |  |
| Meun, 2018 |  |  |  |  |  |  |  |
| Wild, 2000 |  |  |  |  |  |  |  |
| Dahlgren, 1992 |  |  |  |  |  |  |  |
| Iftikhar, 2012 |  |  |  |  |  |  |  |
| Lunde, 2007 |  |  |  |  |  |  |  |
| Schmidt, (2011) |  |  |  |  |  |  |  |
| Shi, 2014 |  |  |  |  |  |  |  |
| Bird, 2012 |  |  |  |  |  |  |  |
| Ramezani Tehrani, 2015 |  |  |  |  |  |  |  |
| Behboudi Gandevani, 2018 |  |  |  |  |  |  |  |
| Definitely No (low risk of bias) probably no  Definitely yes (high risk of bias) probably Yes | | | | | | | |

B.

**Figure S3.** Sensitivity analysis for RR in reproductive age group for all studies.

**Table S4.** sensitivity analysis for RR in reproductive age group for all studies.

**Study omitted | Estimate [95% Conf. Interval]**

Behboudi-Gandevani et al. (2018)| 1.7438776 1.4631997 2.0783963

Bird et al. (2012)| 1.8163466 1.5948005 2.0686698

Calderon-Margalit et al. (2014)| 1.7517917 1.4715889 2.0853477

Chan et al. (2013)| 1.7252944 1.4503318 2.0523858

Chang et al. (2011)| 1.7273015 1.4492077 2.0587597

Chang et al. (2016)| 1.7294208 1.4510241 2.0612314

Dahlgren et al. (1992)| 1.6926078 1.424938 2.0105584

Ding et al. (2018)| 1.7009031 1.4226078 2.0336394

Gateva et al. (2012)| 1.7626334 1.480405 2.0986667

Glintborg et al. (2015)| 1.667802 1.3956908 1.9929655

Haakova et al. (2003)| 1.722587 1.4507524 2.0453565

Hart et al. (2014)| 1.6180612 1.3608732 1.9238546

Iftikhar et al. (2012)| 1.7519197 1.4696397 2.0884185

Li et al. (2013) | 1.7781277 1.4952075 2.1145813

Lo et al. (2006) | 1.6705562 1.3799804 2.0223169

Lunde et al. (2007)| 1.7465718 1.4672309 2.0790954

Luque-Ramirez et al. (2007)| 1.7580776 1.4777819 2.091538

Marchesan et.al (2019)| 1.7026174 1.4301388 2.0270104

Okoroh et al. (2015)| 1.6700399 1.3616711 2.0482428

Ramezani Tehrani et al. (2011)| 1.7311074 1.4567262 2.0571694

Ramezani Tehrani et al. (2014)| 1.7339586 1.4601969 2.059046

Ramezani Tehrani et al. (2015)| 1.7283132 1.4528985 2.0559361

Shi et al. (2014) | 1.7245083 1.4427273 2.0613244

Shroff et al. (2007)| 1.7211879 1.4489206 2.0446172

Sirmans et al. (2014)| 1.6747276 1.399421 2.004195

Vrbíková et al. (2003)| 1.6920248 1.4211771 2.0144906

-------------------+----------------------------------------------------------

Combined | 1.7192039 1.4484419 2.0405803

**Figure S4.** Sensitivity analysis for RR in menopause aging group for all studies.

**Table S5.** Sensitivity analysis for RR in menopause aging group for all studies.

------------------------------------------------------------------------------

Study omitted | Estimate [95% Conf. Interval]

-------------------+----------------------------------------------------------

Behboudi-Gandevani et al. (2018)| 1.3151557 .95511866 1.8109109

Dahlgren et al. (1992)| 1.1271049 .91285157 1.3916451

Merz et al. (2016)| 1.3841956 1.0279515 1.8638988

Meun et. (2018) | 1.3481351 .91374004 1.9890431

Schmidt et al. (2011)| 1.1852014 .87225527 1.6104256

Wild et al. (2000)| 1.3050203 .88736391 1.9192555

-------------------+----------------------------------------------------------

Combined | 1.2624501 .95244965 1.6733485

------------------------------------------------------------------------------

**Figure S5.** Sensitivity analysis for Prevalence in patients with PCOS of reproductive ages.

**Table S6.** Sensitivity analysis for Prevalence in patients with PCOS in reproductive ages.

------------------------------------------------------------------------------

Study omitted | Estimate [95% Conf. Interval]

-------------------+----------------------------------------------------------

Behboudi-Gandevani et al. (2019)|.13882443.10768192.16996695

Bird et al. (2012)| .13402987 .10511241 .16294733

Calderon-Margalit et al. (2014)|.13738079.10572061.16904098

Chan et al. (2013)| .1405253 .10868426 .17236634

Chang et al. (2011)| .13263194 .10089932 .16436456

Chang et al. (2016)| .13359208 .10187376 .16531041

Dahlgren et al. (1992)|.13662526 .10523987 .16801064

Ding et al. (2018)| .1454097 .11199498 .17882441

Gateva et al. (2012)| .13343471 .10178331 .16508612

Glintborg et al. (2015)|.14260861 .11446112 .17075612

Haakova et al. (2003)|.14502619 .11309789 .17695449

Hart et al. (2014)| .14434238 .11196198 .17672278

Iftikhar et al. (2012)|.13321559 .10140928 .1650219

Li et al. (2013) | .13813789 .10620543 .17007037

Lo et al. (2006) | .14011283 .10756706 .17265861

Lunde et al. (2007)| .1413971 .10952041 .1732738

Luque-Ramirez et al. (2007)|.13435052.10286281 .16583823

Marchesan et.al (2019)|.13882443 .10768192 .16996695

Okoroh et al. (2015)| .14271627 .10586881 .17956375

Ramezani Tehrani et al. (2011)|.14476731.11281621 .17671841

Ramezani Tehrani et al. (2014)|.14531368.1133662 .17726114

Ramezani Tehrani et al. (2015)|.14191486.11006496 .17376475

Shi et al. (2014) | .13594127 .10417929 .16770324

Shroff et al. (2007)| .13919833 .10763681 .17075987

Sirmans et al. (2014)|.13232347 .10068957 .1639574

Vrbíková et al. (2003)|.13633762 .10472579 .16794947

-------------------+----------------------------------------------------------

Combined | .13882443 .10768191 .16996694

------------------------------------------------------------------------------

**Figure S6.** Sensitivity analysis for Prevalence in patients with PCOS in menopause aging group.

**Table S7.** Sensitivity analysis for Prevalence in patients with PCOS in menopause aging group.

------------------------------------------------------------------------------

Study omitted | Estimate [95% Conf. Interval]

-------------------+----------------------------------------------------------

Behboudi-Gandevani et al. (2019)|.51420444.26584604.76256281

Dahlgren et al. (1992)|.51026291 .22779246 .79273337

Merz et al. (2016)| .52284539 .23031107 .81537974

Meun et. (2018) | .47367305 .21294805 .73439807

Schmidt et al. (2011)|.47078729 .1944975 .74707705

Wild et al. (2000)| .62384993 .53844118 .70925862

-------------------+----------------------------------------------------------

Combined | .51420442 .26584603 .7625628

------------------------------------------------------------------------------

**Figure S7.** Sensitivity analysis for Prevalence in healthy controls of reproductive ages.

**Table S8.** Sensitivity analysis for Prevalence in healthy control of reproductive ages.

------------------------------------------------------------------------------

Study omitted | Estimate [95% Conf. Interval]

-------------------+----------------------------------------------------------

Behboudi-Gandevani et al. (2019)|.08524524.07134463.09914585

Bird et al. (2012)| .07824372 .06631624 .0901712

Calderon-Margalit et al. (2014)|.08103725.06686623.09520828

Chan et al. (2013)| .08589393 .07173926 .10004859

Chang et al. (2011)| .08233398 .06825068 .09641728

Chang et al. (2016)| .08223884 .0681367 .09634098

Dahlgren et al. (1992)|.0869493 .07281358 .10108502

Ding et al. (2018)| .09043039 .07504443 .10581635

Gateva et al. (2012)| .08085126 .06683648 .09486604

Glintborg et al. (2015)|.09112757 .07550433 .10675082

Haakova et al. (2003)|.08841346 .07419998 .10262694

Hart et al. (2014)| .0911129 .07544126 .10678453

Iftikhar et al. (2012)|.08055217 .06642903 .0946753

Li et al. (2013) | .08148562 .06735143 .09561982

Lo et al. (2006) | .08759554 .07305969 .10213138

Lunde et al. (2007)| .0854909 .07124943 .09973235

Luque-Ramirez et al. (2007)|.08401233.07008898 .09793567

Marchesan et.al (2019)|.08524524 .07134463 .09914585

Okoroh et al. (2015)| .0872822 .0729471 .10161731

Ramezani Tehrani et al. (2011)|.08869115.07441192 .10297038

Ramezani Tehrani et al. (2014)|.08832135.07405568 .10258701

Ramezani Tehrani et al. (2015)|.08702902.07277189 .10128615

Shi et al. (2014) | .08337203 .069165 .09757907

Shroff et al. (2007)| .08526898 .07128266 .09925531

Sirmans et al. (2014)|.08348458 .06935064 .09761851

Vrbíková et al. (2003)|.0854518 .07124299 .09966063

-------------------+----------------------------------------------------------

Combined | .08524524 .07134463 .09914585

------------------------------------------------------------------------------

**Figure S8.** Sensitivity analysis for Prevalence in healthy control of menopause aging group.

**Table S9.** Sensitivity analysis for Prevalence in healthy control of menopause aging group.

------------------------------------------------------------------------------

Study omitted | Estimate [95% Conf. Interval]

-------------------+----------------------------------------------------------

Behboudi-Gandevani et al. (2019)|.40640807.18044683.63236928

Dahlgren et al. (1992)|.4641901 .18987897 .73850125

Merz et al. (2016)| .34890303 .1314237 .56638235

Meun et al. (2018)| .35157928 .11043011 .5927285

Schmidt et al. (2011)|.40541449 .13798599 .67284304

Wild et al. (2000)| .46239108 .25267416 .67210799

-------------------+----------------------------------------------------------

Combined | .40640807 .18044683 .63236931

------------------------------------------------------------------------------

**Figure S9.** The result of sensitivity analysis for all age subgroups.

**Figure S10.** The result of sensitivity analysis for reproductive age subgroup.

**Figure S11.** The result of sensitivity analysis for menopause/aging subgroup.

**Figure S12.** Forest plot of pooled relative risk of HTN for all studies except those with Rotterdam criteria.

**

**Figure S13.** Forest plot of pooled relative risk of HTN for all population based studies except those with Rotterdam criteria.

**

**Figure S14.** Forest plot of pooled relative risk of HTN for all non-population based studies except those with Rotterdam criteria.

**
